# Host switching in a generalist parasitoid: contrasting transient and transgenerational costs associated with novel and original host species

**DOI:** 10.1002/ece3.1333

**Published:** 2015-01-03

**Authors:** Thomas S Jones, Adam R Bilton, Lorraine Mak, Steven M Sait

**Affiliations:** School of Biology, L.C. Miall Building, University of LeedsLeeds, LS2 9JT, U.K

**Keywords:** Adaptation, biocontrol, fitness, invasive species, parasitism, phenotypic plasticity, *Venturia canescens*

## Abstract

Parasitoids face challenges by switching between host species that influence survival and fitness, determine their role in structuring communities, influence species invasions, and affect their importance as biocontrol agents. In the generalist parasitoid, *Venturia canescens* (Gravenhorst) (Hymenoptera: Ichneumonidae), we investigated the costs in encapsulation, survival, and body size on juveniles when adult parasitoids switched from their original host, *Plodia interpunctella* (Hübner) (Lepidotera, Pyralidae) to a novel host, *Ephestia kuehniella* (Zeller) (Lepidoptera, Pyralidae), over multiple generations. Switching had an initial survival cost for juvenile parasitoids in the novel host, but increased survival occurred within two generations. Conversely, mortality in the original host increased. Body size, a proxy for fecundity, also increased with the number of generations in the novel host species, reflecting adaptation or maternal effects due to the larger size of the novel host, and therefore greater resources available to the developing parasitoid. Switching to a novel host appears to have initial costs for a parasitoid, even when the novel host may be better quality, but the costs rapidly diminish. We predict that the net cost of switching to a novel host for parasitoids will be complex and will depend on the initial reduction in fitness from parasitizing a novel host versus local adaptations against parasitoids in the original host.

## Introduction

Parasitoids are a hugely diverse group, in which hymenopteran parasitoids alone are thought to number at least 200,000 species (Pennacchio and Strand 2006). Generalist parasitoids that prey on a range of host species are a key component of many insect communities (Janssen 1989), and the particular host preferences that generalist parasitoids exhibit can have major implications for host population dynamics (Hassell 2000), because they can mediate indirect interactions between host species to determine abundance (Lawton and Strong 1981), coexistence (Bonsall and Hassell 1997), and community structure (van Veen et al. 2006).

If host preferences depend on the relative abundance of different host species, parasitoids, like other generalist predators, exhibit a behavior called “switching” (Murdoch 1969). Theory predicts that switching can arise due to optimal foraging behavior (Křivan 1996), or a preference for parasitizing the same species that a parasitoid developed in, which can generate a population-level switching effect (Hastings and Godfray 1999). Hosts vary in their suitability for parasitoids (Jones et al. 2009), especially because the relationship between an immature endoparasitoid and its host is an intimate one. To survive through to adulthood, the developing parasitoid must avoid or overcome a range of host defenses (Strand and Pech 1995), requiring a high degree of specialization that may limit host range (Van Veen et al. 2008).

Phenotypic plasticity, where an organism is able to express different phenotypes depending on the environment (Agrawal 2001), may enable a parasitoid species to successfully attack a range of host species. There is also growing evidence that a parent's experience of its environment can be passed to its offspring (Mousseau and Fox 1998; Wolf and Wade 2009), so that insects can be “acclimated” to the same host environment in which their parents developed (Fox and Mousseau 1998), which is an example of transgenerational phenotypic plasticity or maternal effects. In the case of parasitoids, offspring would be primed to develop in the host in which their mother developed. This will be adaptive when parental host identity is an indicator of local host availability; however, it may be costly until the parasitoids become acclimated to the changes in the host community if there are rapid changes in relative abundances of hosts, or a novel host establishes in the community (Uller 2008). Alternatively, parasitoids may rapidly adapt to novel host species, although again there may be transient costs until adaptation occurs. This may allow parasitoids to maximize fitness in different host species that each requires different, possibly conflicting, specialist adaptations.

**Figure 1:**
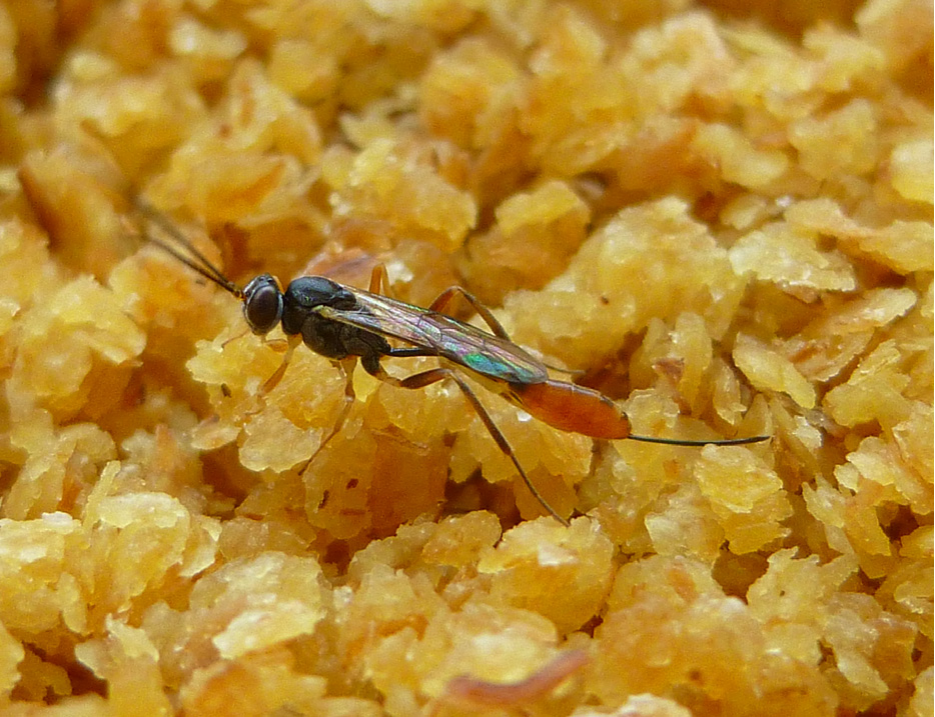
Adult *Venturia canescens* (Gravenhorst) (Hymenoptera: Ichneumonidae), a parasitoid of Lepidoptera (typically Pyralidae). This wasp is from a thelytokous female-only strain, which is typically associated with man-made environments such as grain stores. The adult wasp uses its ovipositor to probe its environment for host larvae, in which to deposit eggs.

Despite the evidence that generalist parasitoids will prey on a range of host species and despite the defense challenge of attacking novel hosts, nothing is known about how the fitness effects of switching partition into costs due to the novelty of the host species, where unfamiliarity with host physiology and defenses reduces parasitoid growth and survival rates, and effects due to host species identity, where different host species are of different inherent value to the parasitoid, effects due to differences in body size, for example. This study examines the costs of switching in the parasitoid *Venturia canescens* (Gravenhorst) (Hymenoptera: Ichneumonidae) (Fig.1), which parasitizes a range of pyralid moths. *V. canescens* is a solitary koinobiont endoparasitoid, which means that a single egg is typically laid inside a host, and a single adult emerges, following a period of delayed development during which the host continues to grow and feed (Godfray 1994). *V. canescens* avoids the host encapsulation response (Kinuthia et al. 1999), can delay its own development, and can regulate host growth (Harvey 1996), all of which maximize adult emergence and fitness, although host species identity strongly influences development and fitness (Harvey and Thompson 1995).

Over three successive *V. canescens* generations, parasitism of an original host, the Indian meal moth *Plodia interpunctella* (Hübner) (Lepidoptera, Pyralidae), and a novel host, the Mediterranean flour moth, *Ephestia kuehniella* (Zeller) (Lepidoptera, Pyralidae), was carried out in order to quantify the potential costs of switching, to determine whether these costs persist over multiple generations and whether adaptation to the novel host can incur fitness costs for future parasitoid generations developing in the original host. To distinguish between intragenerational phenotypic plasticity, transgenerational phenotypic plasticity and/or rapid adaptation, we made the following hypotheses: (1) If intragenerational phenotypic plasticity is the major factor in parasitoid switching, the value of the different host species, in terms of parasitoid development, survival, and fitness, should remain constant over the generations spent in the novel host species. (2) If transgenerational phenotypic plasticity and/or rapid adaptation are the most important factors, we predict that parasitoid fitness will increase in the novel host and decline in the original host, and this process will continue over subsequent generations spent in the novel host.

## Materials and Methods

*Venturia canescens* parasitoids were kept in a controlled environment chamber at 28°C ± 1°C, maintained on final instar *P. interpunctella* larvae in a 0.5 cm depth of wheatgerm-based diet (Niogret et al. 2009), for 18 months. *Venturia canescens* exists with two reproductive modes; as arrhenotokous (sexual) strains, or as parthenogenetic thelytokous (asexual) strains, which are used in this study. Thelytokous wasps produce haploid eggs that undergo diploidy restoration during early embryonic development, so that daughters are not clones of their mother (Beukeboom and Pijnacker 2000).

Newly emerged parasitoids were randomly collected from the laboratory culture and assigned to one of two host treatments for sequential parasitism of the relevant host species. Each parasitoid was placed in a 10-mL clear plastic tube (Starlab, Germany) with one-fifth instar larva of either the novel host *E. kueniella* (EK) or the original host *P. interpunctella* (PI), and they were monitored until a single parasitism was observed. *Venturia canescens* has a distinctive “ovipositor cocking” behavior that confirms oviposition (Rogers 1972). It was not feasible to link individual parent wasps to their offspring during the experiment, but for each individual parasitism, a wasp was randomly selected from an experimental pool to minimize the chance of correlation between experimental treatment and parental wasp identity. After each successful parasitism, the wasp was returned to the experimental pool. If parasitism did not occur within 10 min, the host and parasitoid were discarded. Parasitized hosts were reared individually with excess diet in 25-well petri dishes (Sterilin, U.K.). The parasitoids that emerged – the F1 generation – were then divided into two lines: parasitoids that developed in the novel host EK and parasitoids that continued to develop in the original host PI. Parasitoids from the EK line were randomly assigned to either EK or PI treatments and allowed to singly parasitize larvae as before. Each parasitoid was supplied with fresh hosts unless they did not parasitize a host within 10 min, at which point they were discarded. Parasitized hosts were reared on individually, producing the F2 generation. These steps were repeated to produce the F3 generation.

Parasitized hosts were monitored until either a parasitoid emerged, confirming successful development, or a moth emerged, indicating that the juvenile (egg or larva) parasitoid had been successfully encapsulated, or neither host nor parasitoid survived. Death of the host and parasitoid was due to the juvenile parasitoid consuming the entire host, but failing to complete its own development through lack of sufficient resources (Harvey et al. 1995). The time to adult parasitoid or moth emergence was also recorded. Right hind tibia length was measured on all emerged parasitoids because it is positively correlated with overall body mass and egg load (Harvey et al. 1994). Similar numbers of hosts were parasitized in both host treatments (in the EK treatment, *n* [F1] = 30, *n* [F2] = 29, and *n* [F3] = 29; in the PI treatment, *n* [F1] = 30, *n* [F2] = 27, and *n* [F3] = 29); the probability of successful parasitism within 10 min was not affected by host species, but did increase over generations (Fig. S1).

Encapsulation rates and parasitoid emergence rates were analyzed with generalized linear models specifying binomial errors, with offspring generation, host species, and the interaction fitted as fixed effects. Stepwise model simplification and chi-squared tests were used to determine the significance of fixed effects (Crawley 2007). Hind tibia length was analyzed using ANCOVA with offspring generation, host species, and the interaction fitted as fixed effects. *F*-tests were used to determine the significance of fixed effects.

## Results

### Encapsulation

**Figure 2:**
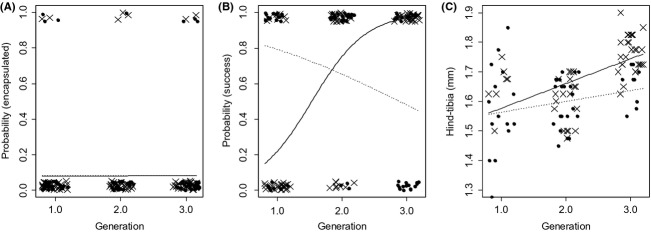
*Venturia canescens* encapsulation rate (A), successful development rate (B), and hind tibia length (C) when parasitizing *E. kuehniella* (solid lines, crosses) and *P. interpunctella* (dashed line, solid points), after *V. canescens* had 0,1, or 2 previous generations developing in *E. kuehniella*. Offspring generation was fitted as continuous variable in all analyses following (Gelman and Hill 2007). The lines on each graph are regression lines of the response against offspring generation. In the case of encapsulation rate and successful development rate, the model coefficients were inverse-logit transformed prior to drawing the regression lines.

Eight percent of all hosts encapsulated their parasitoids, indicating a weak immune defense against *V. canescens* in both host species, which is consistent with how *V. canescens* avoids the host's immune system (Kinuthia et al. 1999) (Fig.2A). We found no difference in encapsulation rate between host species (logistic regression, host effect 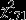
_ _= 0.002, N.S.), and the number of previous generations the parasitoid had parasitized *E. kuehniella* did not affect encapsulation rate in either host species (logistic regression, generation 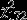 = 0.003, N.S., host–generation interaction 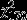 = 0.00, N.S.)

### Emergence rate

Of the juvenile parasitoids that were not encapsulated, the proportion that successfully emerged in *E. kuehniella* increased dramatically from 25% in the F1 generation to 100% in the F3 generation. In contrast, surprisingly, the proportion that developed and emerged successfully in *P. interpunctella* fell from 67% in the F1 generation to 36% in the F3 generation (Fig.2B, logistic regression, host–generation interaction 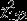 = 40.0, *P *<* *0.001). Hence, the original host became much less suitable once the parasitoid had switched. There was no effect of host species or number of generations on development time (Fig. S2).

### Body size

Parasitoids that developed in *P. interpunctella* were smaller than individuals that developed in *E. kuehniella* (hind tibia length, Fig.2C, ANCOVA, host *F*_1,95_ = 9.08, *P *=* *0.003). Size increased over parasitoid generations (Fig.2C, ANCOVA, generation *F*_1,95_ = 31.6, *P *<* *0.001), but there was no difference in the size increase between parasitoids that developed in *P. interpunctella* and *E. kuehniella* (ANCOVA, host–generation interaction *F*_1,95_ = 2.67, N.S.).

## Discussion

Switching to a novel host species caused a large initial reduction in survival through to adulthood in the parasitoid *Venturia canescens*, but survival increased dramatically over three successive generations in the novel host species. However, this response to the novel host was accompanied by a cost in terms of reduced survival to emergence in the original host species. This supports our hypothesis that transgenerational phenotypic plasticity and host-specific adaptation, rather than intragenerational phenotypic plasticity, were major factors determining parasitoid fitness when switching to a novel host.

Encapsulation rates were consistently low in both host species regardless of host novelty or number of generations, and this reflects the fact that *V. canescens* is able to go undetected by the host's immune system by utilizing host proteins as a protective barrier (Kinuthia et al. 1999). Hence, a generalist parasitoid that does not rely on countermeasures targeted at a specific host species may be able to avoid encapsulation or other immune defenses in a broader range of host species, which may otherwise be a barrier to host switching.

Parasitoid emergence in the first generation was higher in the original host, *P. interpunctella,* than in the novel host, *E. kuehniella*. The factor affecting parasitoid emergence rates was whether the parasitoid successfully completed development, rather than whether it avoided encapsulation. This was due to the larval parasitoid consuming its host before completing its own development and then starving to death. This may be because it did not respond correctly to host cues. This finding concurs with previous studies, where *V. canescens* emergence was higher in the original host for two different combinations of original and novel hosts (Harvey and Thompson 1995; Harvey and Vet 1997), suggesting that the parasitoid lacks the intragenerational phenotypic plasticity to optimize development in different species of host simultaneously. However, neither of these studies examined and contrasted juvenile parasitoid survival in the novel and original host species in subsequent generations. We found that emergence rates in the novel host increased dramatically, while emergence rates in the original host fell in the second and third generations, suggesting that host familiarity could be more important than host suitability *per se* in determining parasitoid emergence.

These changes may have occurred due to rapid adaptation to the novel host, a phenomenon observed in the seed beetle *Callosobruchus maculatus*, which adapted to a novel food source over 35 generations via changes to oviposition behavior (Fricke and Arnqvist 2007). In our study, host species identity did not affect host acceptance by the parasitoid (Fig. S1), adaptation was primarily postoviposition, and occurred over three generations. Spider mites also exhibit rapid, local adaptation to different host plant species, but in contrast to this study, there were no adaptation costs (Magalhaes et al. 2009). The extreme costs in terms of mortality apparent in this study may be due to the particularly intimate interaction between parasitoids and their hosts for which survival is all-or-nothing. Alternatively, juvenile parasitoids in generation F2 onward may have been primed to develop in the novel host using cues from the maternal developmental environment, while losing the cues from the original host (Mousseau and Fox 1998). This may suggest transgenerational phenotypic plasticity (Uller 2008), but evidence for this process from experimental studies in similar systems is mixed (Via 1991; Fox et al. 1995; Henniges-Janssen et al. 2011).

Parasitoids that emerged from *E. kuehniella* were larger compared to those that emerged from *P. interpunctella,* a relationship that held across all generations. This reflected host size differences. Fifth instar *E. kuehniella* are substantially larger than fifth instar *P. interpunctella* (*E. kuehniella *= 25.8 mg, *P. interpunctella *= 22.6 mg), and host size is a reliable indicator of the size of the parasitoid that will emerge (Harvey et al. 1994). Clearly, the novel host provides enough resources for the juvenile parasitoid to complete its own development, which makes the initial high levels of parasitoid mortality even more surprising. One possible explanation is that the decision to begin precocious juvenile development (Harvey et al. 1994) is mis-timed in the novel host and it may begin consumption before the host has reached its optimal size. In subsequent generations, juvenile parasitoid mortality in the original host increased, suggesting that as the parasitoid adapts or acclimates to the novel host, it loses the ability to respond to cues in the original host.

Hind tibia length increased across generations in adults emerging from both host species. *E. kuehniella* is larger than *P. interpunctella*, and the nutritional environment of a parent can influence offspring growth and reproductive potential through egg provisioning (Giron and Casas 2003), or through maternal cues (Mousseau and Fox 1998).The observed size increase in *P.interpunctella* over generations may be due to the beneficial effects of the maternal and grandmaternal generations developing in *E. kuehniella*. Alternatively, if larger individuals were more likely to survive developing in a novel host, and assuming a heritable component to body size, the observed size changes over generations may reflect selection for increased body size.

Given the large initial costs of switching to the novel host, we predict that parasitoids will tend to show a preference for ovipositing in the host species in which they developed regardless of host identity and the availability of alternative hosts, which may lead to more limited prey switching by generalist predators. Much theory has explored the role of natural enemies attacking alternative host/prey species (e.g., (Murdoch 1969; Holt 1977)), but little theory about parasitoid–multiple host interactions has included costs for switching between host species (Hassell 2000). However, these costs, and the time frame over which they are incurred, may influence host selection behavior and host-parasitoid dynamics in multispecies communities. Incorporating these costs into host–parasitoid models may provide important insights into when switching between prey species should occur, natural enemy–prey dynamics, and the role of natural enemies in structuring multispecies communities (van Veen et al. 2006).

Recent studies have constructed quantitative food webs to document the strength of links between hosts and parasitoids in natural communities [e.g., (Memmott et al. 1994; Muller et al. 1999)], which highlight the potential importance of parasitoid-mediated indirect interactions between host species that may determine community structure. However, the majority of these studies are observational, estimating link strength based on the numbers of parasitoids emerging from different host species. The results above suggest that links between host species mediated by parasitoids may be limited by the short-term costs of switching between host species. As well as influencing host–parasitoid food web structure, this may have major implications for the use of particular parasitoid populations that are assumed to be more adapted or acclimated to particular pests as part of an optimal biocontrol strategy (Henry et al. 2010). The extent to which parasitoids, or natural enemies more generally, switch to novel prey and effectively limit their numbers may play a key role in invasion, whether an invasive enemy switches to native prey or resident enemies switch to invasive prey (Gripenberg et al. 2011; Jaworski et al. 2013). Of potential concern may be the conventional practice in classical biological control of releasing exotic parasitoid species to control invasive pests following native host range trials that typically only assess host suitability in a single assay (van Lenteren et al. 2006). Our results suggest that such trials may be misleading.

## Supporting Information

**Figure S1.** Probability that *V. canescens* parasitizes a host within 10 min when the host is *E. kuehniella* (solid lines, crosses), or *P. interpunctella* (dashed line, empty points), after *V. canescens* had 0, 1, or 2 previous generations developing in *E. kuehniella* as opposed to *P. interpunctella*. **Figure S2.***Venturia canescens* time to emergence when parasitizing *E. kuehniella* (solid lines, crosses), and *P. interpunctella* (dashed line, filled points), after *V. canescens* had 0,1, or 2 previous generations developing in *E. kuehniella* as opposed to *P. interpunctella*. ANCOVA: generation *F*_1,98_ = 2.41, N.S., host *F*_1,97_ = 1.05, N.S., generation-host interaction *F*_1,96_ = 0.00, N.S.
