# Supplementary material for: Host switching in a generalist parasitoid: contrasting transient and transgenerational costs associated with novel and original host species
Source: Ecol Evol. 2015 Jan 3;5(2):459–65. doi: 10.1002/ece3.1333 (PMC4314276; doi:10.1002/ece3.1333)
Supplement: Supplementary file 1 [file ece30005-0459-sd1.docx]

**Supplementary information.**

**Figure S1.** Probability that *V. canescens* parasitizes a host within 10 minutes when the host is *E. kuehniella* (solid lines, crosses), or *P. interpunctella* (dashed line, empty points), after *V. canescens* had 0,1, or 2 previous generations developing in *E. kuehniella* as opposed to *P. interpunctella*. Logistic regression: generation χ ^2^ _1,226_ = 36.6, P <0.001, host χ ^2^ _1,225_ = 0.75, N.S., generation-host interaction χ ^2^ _1,224_ = 0.00, N.S.

**Figure S2.** *V. canescens* time to emergence when parasitizing *E. kuehniella* (solid lines, crosses), and *P. interpunctella* (dashed line, filled points), after *V. canescens* had 0,1, or 2 previous generations developing in *E. kuehniella* as opposed to *P. interpunctella*. ANCOVA: generation F_1,98_ = 2.41, N.S., host F_1,97_ = 1.05, N.S., generation-host interaction F_1,96_ = 0.00, N.S.
